# Remarkable Response to Etoposide and Cisplatin in Aggressive-Variant Prostate Cancer With Low Prostate-Specific Antigen Levels: A Case Report

**DOI:** 10.7759/cureus.100713

**Published:** 2026-01-03

**Authors:** Junichi Ikeda, Hisanori Taniguchi, Monta Inoue, Yuki Masuo, Takahiro Nakamoto, Masaaki Yanishi, Katsunori Uchida, Hidefumi Kinoshita

**Affiliations:** 1 Urology and Andrology, Kansai Medical University, Hirakata, JPN; 2 Pathology, Kansai Medical University, Hirakata, JPN

**Keywords:** aggressive-variant prostate cancer, cisplatin, etoposide, prostate-specific antigen, radium-223

## Abstract

Prostate-specific antigen (PSA) is a cornerstone of screening for prostate cancer (PC); however, serum PSA levels may remain deceptively low in certain high-grade, aggressive subtypes. Aggressive-variant prostate cancer (AVPC) is a clinical entity characterized by rapid progression, bulky lymphadenopathy, and visceral metastases despite low or non-correlative PSA levels, often requiring treatment approaches beyond conventional therapies. Here, we report the case of a 69-year-old Japanese man with multiple comorbidities, including diabetes, hyperlipidemia, hypertension, angina pectoris, and lower limb occlusive arteriosclerosis, who presented to our center with lower back pain and gait disturbance. Diagnostic imaging demonstrated extensive pelvic bone metastases, seminal vesicle invasion, and pelvic and para-aortic lymphadenopathy. Laboratory evaluation showed a normal PSA level of 2.95 ng/mL and an elevated alkaline phosphatase (ALP) of 669 U/L. A prostate biopsy revealed acinar adenocarcinoma with a Gleason score of 8 (4+4) and intraductal features. Immunohistochemical tests showed positive results for NKX3.1 and androgen receptor but negative results for PSA, synaptophysin, and chromogranin A. Given these findings, the patient was diagnosed with AVPC (cT3bN1M1b) and underwent androgen deprivation therapy combined with etoposide and cisplatin (EP) chemotherapy, achieving a partial response with marked improvements in his symptoms and lymph node lesions. Subsequent radium-223 therapy further reduced bone metastases and normalized ALP levels, leading to substantial functional recovery. This case underscores the necessity of maintaining a high index of suspicion for AVPC when clinical findings contradict low PSA values. Furthermore, the favorable response to EP highlights the potential role of platinum-based chemotherapy in managing low-PSA, high-grade PC. Additional cases are needed to refine the clinical characterization of AVPC and establish evidence-based treatment guidelines.

## Introduction

Prostate-specific antigen (PSA) is a marker widely used to screen for and monitor prostate cancer (PC). Although PSA levels exceeding 4.0 ng/mL have conventionally been interpreted as an indicator of malignant tumors, PC can still be diagnosed when PSA levels are 4.0 ng/mL or lower [1]. Among men with low PSA levels, PC is detected in approximately 15.2% of cases, the majority of which are low-grade with a Gleason score of 6. However, high-grade PC can also present with PSA levels below 4.0 ng/mL, posing a significant diagnostic and therapeutic challenge [2]. There is no established treatment protocol for high-grade PC with low PSA levels, but radical prostatectomy or radiation therapy is typically performed if there is no evidence of distant metastasis [3].

Aggressive-variant prostate cancer (AVPC) is a clinically aggressive form of PC characterized by rapid progression, low or discordant PSA levels relative to tumor burden, androgen receptor (AR) pathway independence, visceral or bulky disease, and a tendency to respond to platinum-based chemotherapy [4,5]. In this case report, we describe a patient with AVPC and extensive bone metastases despite a low PSA level, in whom combination therapy with etoposide and cisplatin (EP) yielded a remarkable clinical and radiographic response.

## Case presentation

A 69-year-old Japanese man with a history of diabetes, hyperlipidemia, hypertension, angina pectoris, and lower limb occlusive arteriosclerosis presented to the orthopedic department of our center with complaints of lower back pain and difficulty walking. Myelography revealed bone metastasis, prompting referral to our Department of Urology for suspected advanced PC. Initial laboratory workups are shown in Table 1.

**Table 1:** Laboratory workup of the patient.

Laboratory test	Result	Normal range
Prostate-specific antigen (ng/mL)	2.95	0.0–4.0
Neuron-specific enolase (ng/mL)	10.3	0.0–16.3
Pro-gastrin-releasing peptide (pg/mL)	112.8	0.0–81.0
Alkaline phosphatase (U/L)	669	38–113

**Figure 1:**
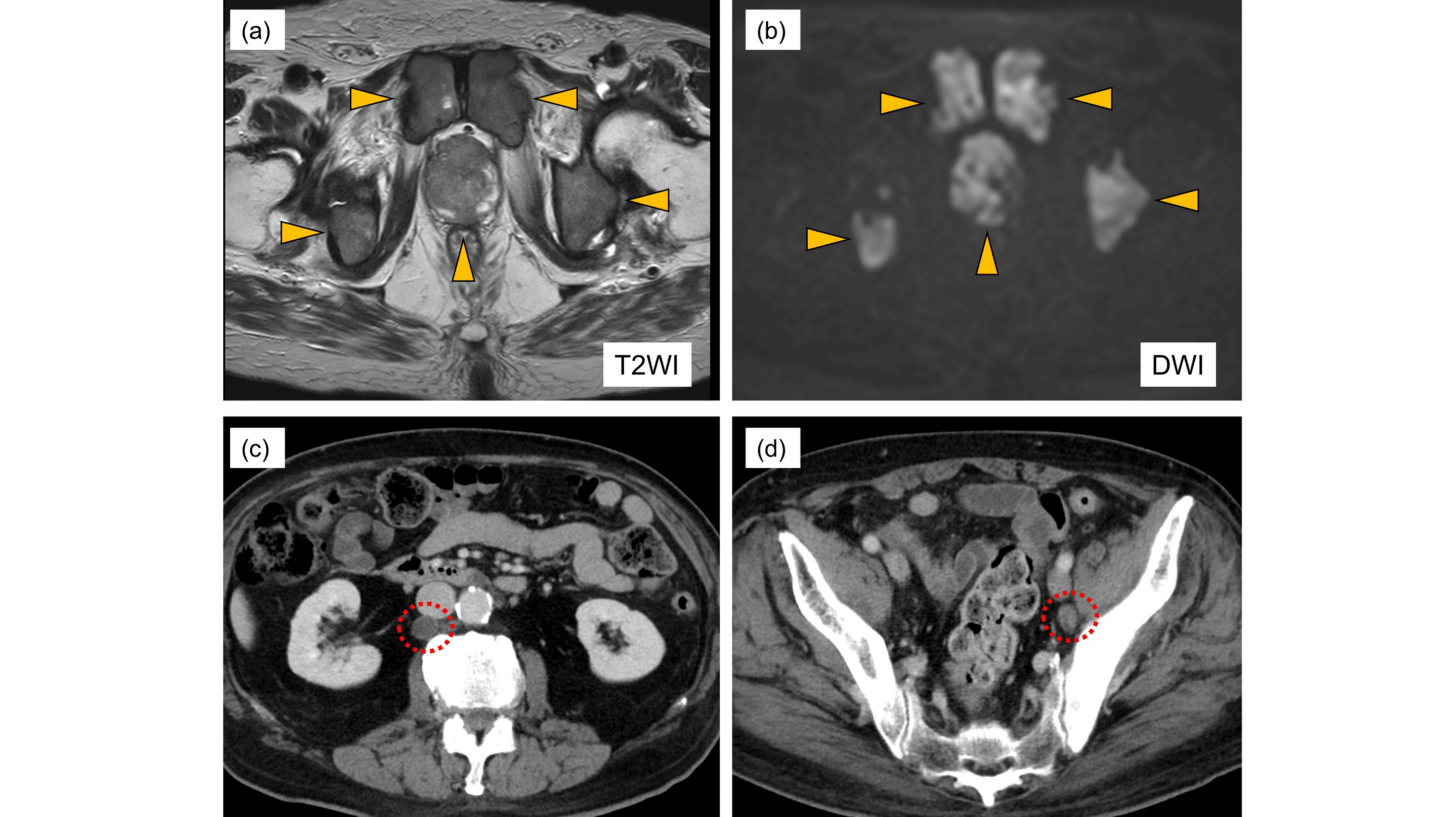
MRI and contrast-enhanced CT images. (a, b) MRI shows an extensive prostate tumor with multiple pelvic bone metastases. The arrows indicate the bone metastasis. (c) Contrast-enhanced CT demonstrates enlargement of para-aortic lymph nodes. (d) Contrast-enhanced CT reveals enlargement of the left internal iliac lymph nodes. The circles indicate the lymph node metastasis.

Digital rectal examination identified a palpable mass extending beyond the prostate, and MRI showed prostate tumor invasion into the seminal vesicle (Figure 1).

**Figure 2:**
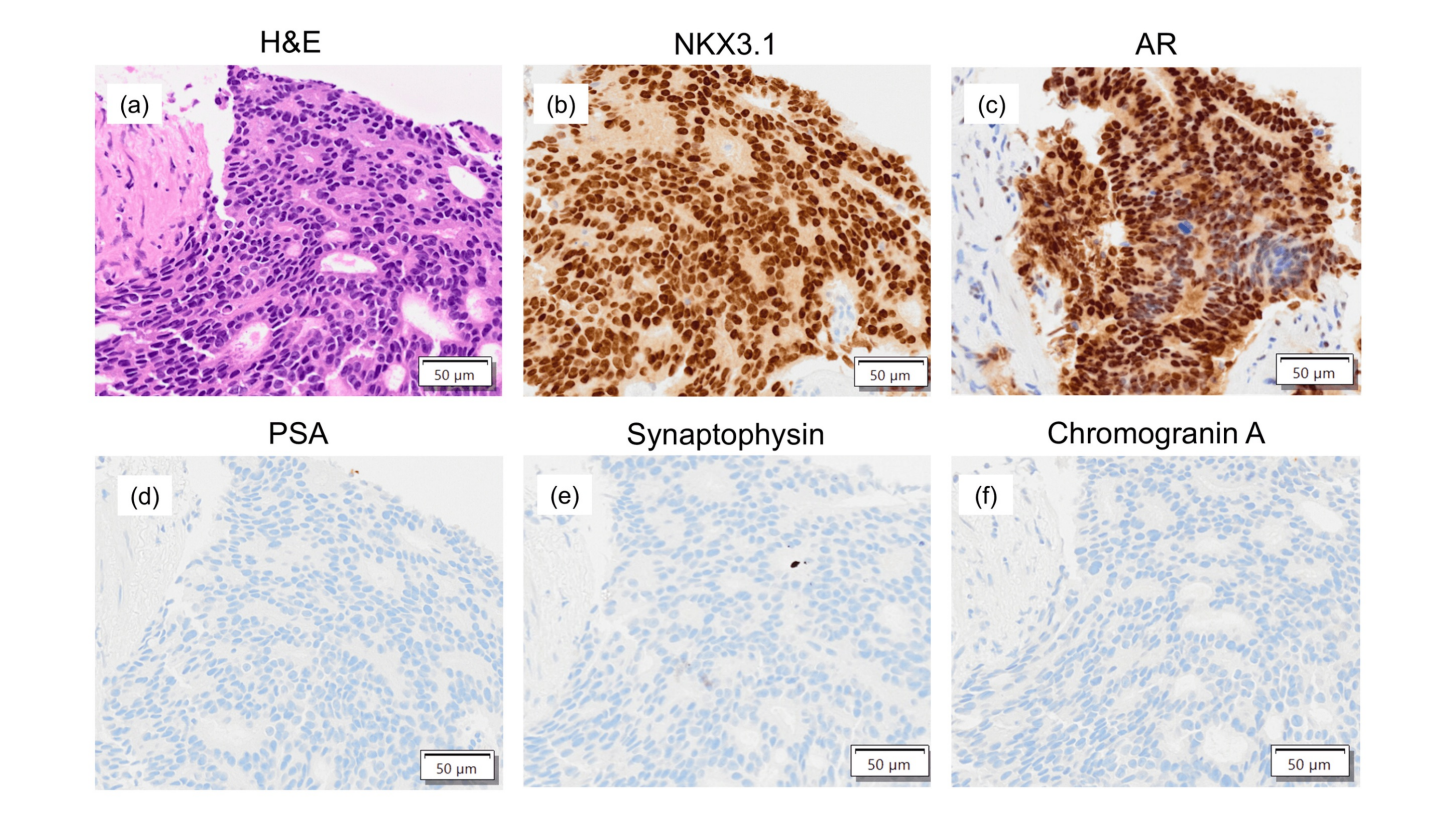
Pathological findings. (a) Hematoxylin and eosin staining shows adenocarcinoma with a Gleason score of 4+4=8. Immunohistochemical analysis shows positive results for (b) NKX3.1 and (c) androgen receptor (AR) but negative results for (d) prostate-specific antigen (PSA), (e) synaptophysin, and (f) chromogranin A.

A CT scan showed metastasis to the left obturator and para-aortic lymph nodes (Figure 1). A subsequent prostate needle biopsy established a pathological diagnosis of prostate adenocarcinoma with a Gleason score of 4+4=8 and intraductal components. Immunohistochemical tests showed positive results for NKX3.1 and AR but negative results for PSA, synaptophysin, and chromogranin A (Figure 2).

**Figure 3:**
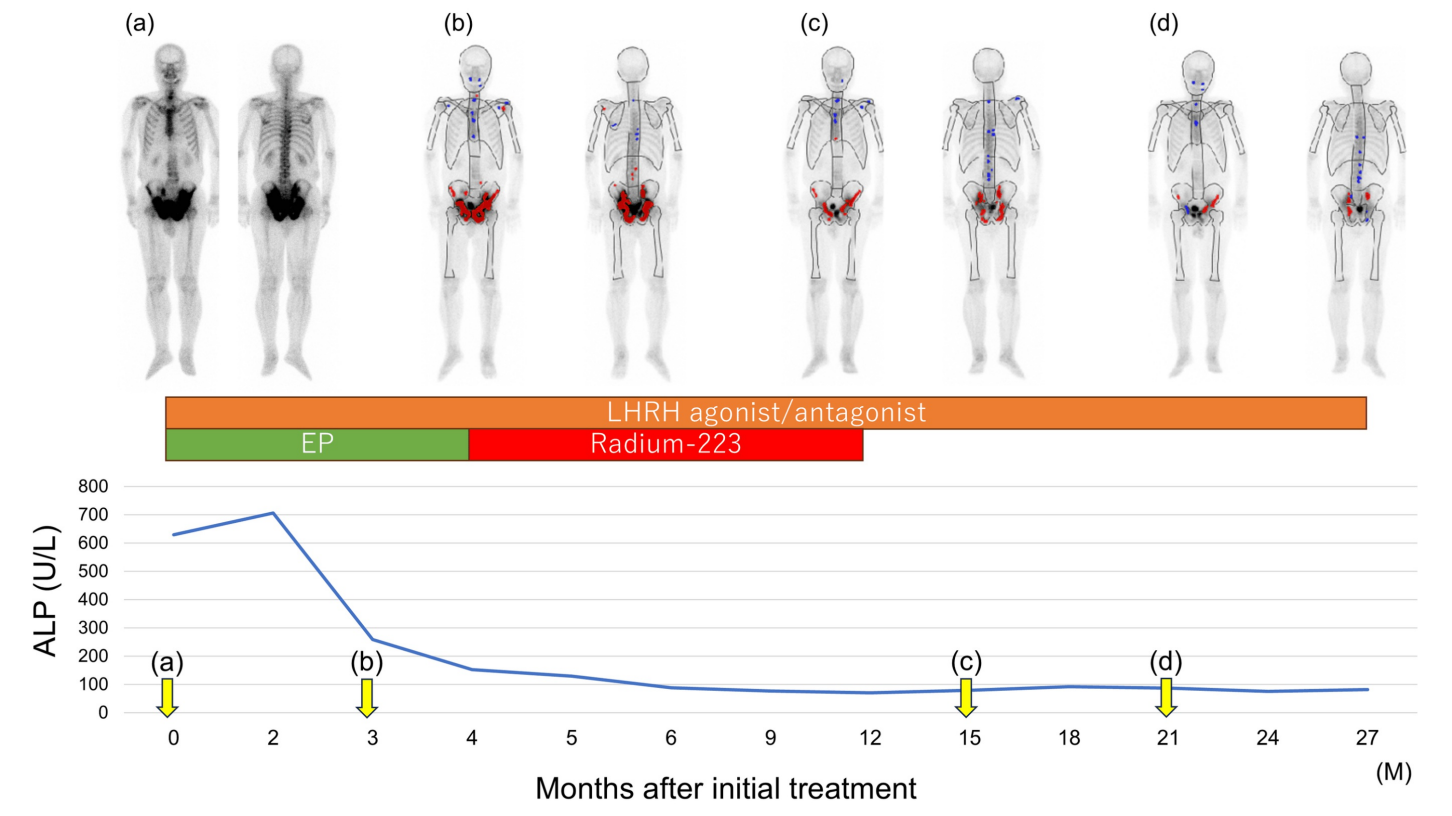
Course of treatment. Alkaline phosphatase (ALP) levels decreased within two months after treatment initiation and have remained normal thereafter. Bone metastases progressively regressed during therapy. The timing and images of the bone scans are shown in a-d.

The Ki-67 proliferation index was 30%. Bone scintigraphy showed widespread radiotracer uptake in the pelvic bone, suggesting high-volume osseous metastases (Figure 3). The disease was staged as cT3bN1M1b. Therefore, the patient was started on combined androgen deprivation therapy (with a luteinizing hormone-releasing hormone antagonist) and EP therapy. The EP regimen consisted of intravenous etoposide (100 mg/m² daily for three consecutive days) and cisplatin (80 mg/m² on day one and repeated every three or four weeks). After four cycles of EP therapy, a partial response was achieved, with resolution of the lymph node lesions; only the bone metastases persisted. The patient subsequently received six courses of radium-223 therapy, which resulted in the shrinkage of the bone metastases, normalization of alkaline phosphatase levels, and improvement of his walking ability (Figure 3).

## Discussion

PSA testing is used to screen for PC [6]. Although PSA levels exceeding 4.0 ng/mL are highly suggestive of malignancy, PC cases can still occur at lower PSA levels. Indeed, previous research indicates that PC is detected in 15.2% of men with PSA levels ≤4.0 ng/mL [1]. In most of these low-PSA PC cases, tumors are low-grade; however, a subset of patients present with high-grade disease, such as AVPC with a Gleason score of 8 or higher. AVPC is clinically characterized by one or more of the following features: presence of visceral metastases, predominantly lytic bone lesions, bulky lymphadenopathy, low or modest PSA levels relative to disease burden, rapid disease progression, and resistance to AR-targeted therapy [4]. Most cases of neuroendocrine prostate cancer (NEPC) fall within the spectrum of AVPC. While the proportion of AVPC among de novo cases remains unknown, de novo NEPC is estimated to account for fewer than 2% of cases [7]. In the case presented here, the combination of a low PSA value and extensive bone metastases led to the diagnosis of AVPC.

PSA expression has been linked to AR activity [8], with lower PSA levels suggesting diminished AR activity in patients with PC [9]. AR staining reduction of less than 10% was observed in 36% of AVPC cases [4]. It has been reported that high-grade PC with low PSA levels tend to progress rapidly and exhibit resistance to hormone therapy [10]. While taxane-based chemotherapy regimens are the standard treatment for metastatic hormone-sensitive PC and docetaxel has shown efficacy in low-PSA disease [11], AVPC possesses a distinct vulnerability to platinum agents [5,12]. Clinical trials have demonstrated favorable outcomes with platinum-based chemotherapy, including combination therapy with cabazitaxel and carboplatin or EP therapy following carboplatin and docetaxel administration [5,12]. In our case, EP was initiated as frontline chemotherapy, eliciting a partial response.

AVPC has been associated with aberrations in multiple tumor suppressor genes, including *RB1*, *TP53*, and/or *PTEN* [4]. These genetic abnormalities lead to DNA repair deficiencies, which, in turn, confer sensitivity to platinum-based chemotherapy. In our case, immunohistochemical analysis demonstrated positive AR expression, indicating residual hormone responsiveness. Therefore, androgen deprivation therapy was administered in addition to platinum‑based treatment.

In cases of PC showing progression despite low PSA levels, it is important to suspect the presence of AVPC at an early stage. The clear tumor shrinkage observed from the outset with EP therapy in this case suggests that platinum-based combination chemotherapy may represent an effective treatment option against PSA-independent disease progression. Future studies are needed to further define the clinical and histological characteristics of AVPC and optimize regimen selection.

## Conclusions

EP therapy may represent an effective treatment option for AVPC presenting with low PSA levels; however, larger case series and prospective studies are needed to better define its therapeutic role.
